# Ethical dilemmas are really important to potential adopters of autonomous vehicles

**DOI:** 10.1007/s10676-021-09605-y

**Published:** 2021-07-02

**Authors:** Tripat Gill

**Affiliations:** Lazaridis School of Business & Economics, Wilfrid Laurier University, Waterloo, ON Canada

**Keywords:** Ethical dilemmas, Autonomous vehicles, Innovation adoption, Risk, Technology

## Abstract

The ethical dilemma (ED) of whether autonomous vehicles (AVs) should protect the passengers or pedestrians when harm is unavoidable has been widely researched and debated. Several behavioral scientists have sought public opinion on this issue, based on the premise that EDs are critical to resolve for AV adoption. However, many scholars and industry participants have downplayed the importance of these edge cases. Policy makers also advocate a focus on higher level ethical principles rather than on a specific solution to EDs. But conspicuously absent from this debate is the view of the consumers or potential adopters, who will be instrumental to the success of AVs. The current research investigated this issue both from a theoretical standpoint and through empirical research. The literature on innovation adoption and risk perception suggests that EDs will be heavily weighted by potential adopters of AVs. Two studies conducted with a broad sample of consumers verified this assertion. The results from these studies showed that people associated EDs with the highest risk and considered EDs as the most important issue to address as compared to the other technical, legal and ethical issues facing AVs. As such, EDs need to be addressed to ensure robustness in the design of AVs and to assure consumers of the safety of this promising technology. Some preliminary evidence is provided about interventions to resolve the social dilemma in EDs and about the ethical preferences of prospective early adopters of AVs.

**Supplementary Information:**

The online version contains supplementary material available at 10.1007/s10676-021-09605-y.

*“There are two possible ways to approach phenomena. The first is to rule out the extraordinary and focus on the "normal." The examiner leaves aside "outliers" and studies ordinary cases. The second approach is to consider that in order to understand a phenomenon, one needs to first consider the extremes - particularly if, like the Black Swan, they carry an extraordinary cumulative effect.”* (Taleb, 2007).

## Introduction

Autonomous vehicles (AVs) promise tremendous benefits to consumers and to society at large (e.g., reduced pollution and congestion, and a major reduction in traffic accidents; Waldrop, 2015). The commercialization of AVs is considered a moral imperative if it can save thousands of lives and is predicted to be a US$7 trillion market by 2050 (Lanctot, 2017). However, consumers remain skeptical about AVs. For instance, a recent survey found that nearly 75% of Americans fear riding in a self-driving car (Edmonds, 2019). Among the many adoption barriers to AVs, a major psychological roadblock may be the perceived risk in assigning control to an autonomous agent (Shariff et al., 2017). This risk is especially apparent when AVs would need to make moral decisions that entail harm to either the passengers or other parties on the road. For instance, should AVs protect the lives of passengers or that of the pedestrians; should they preferentially save the young over the old, when harm is unavoidable?

These ethical dilemmas (EDs) for AVs have captured the imagination of academic researchers, legal experts and policy makers, and have also gained widespread media attention. Several large-scale behavioral studies using such EDs—modelled after the classic *trolley problem* (Foot, 1967; Thomson, 1985) in moral psychology—have been published in prestigious academic outlets (e.g., Awad et al., 2018; Bonnefon et al., 2016). These studies are based on the premise that EDs are extremely important to address for consumer acceptance of AVs, and the solution to these dilemmas is best determined by seeking public opinion on this matter (Awad et al., 2020a, b; Rahwan, 2018). Moreover, not addressing this issue could lead to an ethical opt-out problem (Bonnefon et al., 2020a). Consumers would just not adopt AVs that fail to meet their ethical expectations about whom to protect when harm is unavoidable.

However, the approaches that assess AV ethics using the trolley-type dilemma has its detractors. Some prominent academic scholars have decreed that EDs are essentially an engineering and policy distraction, and not the most pressing issues for AVs (De Frietas et al., 2020, 2021; Dewitt et al., 2019; Nyhoml & Smids, 2016). They argue that such EDs are too contrived, highly unlikely, and difficult for AVs to detect or solve if they were to occur. Moreover, they contend that other technological problems (e.g., ability to operate in inclement weather, recognize objects, and accurately read road signage) are more pressing issues. The AV designers and manufacturers also concur with this view and do not consider EDs as an issue for them to solve (Iagnemma, 2018; Olson, 2018). The recent European Commission report on the Ethics of Connected and Automated Vehicles (CAV) contends that “moral dilemmas in crash avoidance are not the only, nor even the most urgent, ethical and societal issue raised by CAV safety” (p. 17; Bonnefon et al., 2020b; referred to as CAV2020 from hereon).

While both sides of this debate speculate upon the significance of EDs, the opinion of the most critical stakeholder—the potential adopters^1^ of AVs—has been ignored. Namely, what do consumers think about the importance of addressing EDs for the adoption of self-driving cars? Furthermore, how are these EDs perceived *relative* to the other challenges facing AVs. What is the relative significance of the EDs compared to (a) the technical/engineering issues (e.g., accurately recognizing signs and objects, availability of high-quality maps, and the effect of inclement weather), (b) the legal challenges (e.g. who is liable for the harm caused by AVs), and (c) other ethical issues (e.g., data privacy) for AVs? If consumers view the EDs as relatively unimportant in their decision to adopt AVs, then indeed it is a waste of academic, government, and industry resources in addressing this issue. On the other hand, if consumers view that it is critically important to address such dilemmas for AVs, then research on this issue is worthwhile and government regulators need to put efforts into resolving them.

The current article offers the potential AV adopters’ perspective on these issues. Specifically, it (1) draws on the literature on innovation adoption to highlight the role of *perceived risks* in the adoption of AVs, (2) discusses how consumers may assess the risks posed by EDs, and (3) provides empirical evidence for the *relative* importance that people assign to overcoming EDs versus the other challenges to the adoption of AVs. Overall, the established research on innovation adoption and risk perception suggests that the EDs would be associated with substantial risk and perceived as a significantly important challenge to overcome for the adoption of AVs. Empirical evidence obtained from two studies using a quasi-representative sample of US participants (*N* = 1006) and from undergraduate students (*N* = 672) supported this assertion. Specifically, the ED of whom to protect—passenger or pedestrian—if harm is unavoidable was not only perceived as a critically important issue for adopters, but it was also considered significantly more important than any of the technical, legal or other ethical challenges facing AVs. This result was not influenced by prior exposure to AV dilemmas and nor by demographic or behavioral factors. In sum, EDs do not seem a distraction from the adopters’ perspective. But rather they appear to be the most critical issue to reduce consumer apprehension about AVs.

The rest of the article is organized as follows. First, the benefits of AVs and the technical, legal and ethical challenges to their adoption are discussed. Then the research and debate pertaining to EDs in the context of AVs is briefly summarized. Thereafter, research on innovation adoption and risk perception is used to examine the consumer’s perceived importance of the different challenges facing AVs. Then two empirical studies are presented, which investigated the relative importance and risks perceived by consumers in the EDs versus the other issues pertaining to AV adoption. Finally, implications of these findings for academic researchers, AV manufacturers and policy makers are discussed. Also, some preliminary evidence is presented about the ethical preferences of the likely early adopters of AVs, and avenues for future research are suggested.

## The benefits and challenges in the adoption of AVs

Every year close to 1.25 million people die in traffic accidents, more than 90% of which are said to be caused by human error (Singh, 2015; Waldrop, 2015). AVs or self-driving cars are being promoted as an effective solution to these problems, as they are not prone to the most common human errors that cause accidents (e.g., distracted or aggressive driving, intoxication, and fatigue) (Miller, 2014; Deng, 2015; Woodyard, 2015; Saunders, 2020). AVs would also help in reducing congestion on the roads and lower pollution (Pyper, 2014). Concurrently, decreased traffic accidents by AVs will reduce costs on the health sector (Rojas-Rueda et al., 2020), and decrease costs on vehicle insurance (Light, 2012). AVs will relieve travellers from driving tasks thereby providing more time for leisure and work during commutes (Cowen, 2013; Deng, 2015). In addition, several in-car entertainment and information services could enhance the travel experience of AV users (Lanctot, 2017). For the young, elderly and people with disabilities, AVs can provide superior options for mobility (Halsely III, 2017). AVs and autonomous taxis could also lessen the need for parking spaces and help reclaim land use in urban areas (Stewart, 2018). As such, several automobile manufacturers and technology companies are banking on the promising future of AVs and have devoted significant resources in developing such vehicles.

Despite their tremendous benefits, the adoption of AVs is not expected to be straightforward. Recent surveys show that three out of four consumers are skeptical about riding in AVs (Edmonds, 2019). There are indeed several barriers to the consumer adoption of AVs, including technical, legal and ethical challenges. Technologically, the artificial intelligence underlying AVs is still not able to function well in chaotic inner-city environments (Gomes, 2014); the car’s sensing and navigation systems are susceptible to bad weather conditions (such as rain and snow) (Tussy, 2016); the object recognition system cannot accurately read all road signs, recognize large animals and other unusual obstacles (Zhou, 2017); AVs need very high quality specialized 3-dimensional maps to operate properly and some of the current road infrastructure may need to be modified for AVs to function optimally (Badger, 2015); and AVs cannot fully understand the gestures and non-verbal cues of other drivers and pedestrians (Gomes, 2014).

AVs also face a slew of legal and regulatory issues that can hinder their adoption. There is a need to determine who is liable when an AV causes physical damage to people, other vehicles, or breaks traffic rules (Strong & Baker, 2015). As the control over driving operations shifts from humans to AV technology, the current liability laws need to be modified, and the responsibility for damages needs to be determined for the passengers, AV manufacturers and insurers (Vladeck, 2014). As such, several policies and regulations pertaining to the legal issues around AVs are being drafted across the world (e.g., see Luetge, 2017 for the German ethics code for connected and automated driving, and CAV2020 for the regulatory policies recommended by the European Commission.).

AVs introduce some unique ethical challenges in their operation. One such ethical issue that has garnered the most attention among academic researchers is the ED: namely, the dilemma between saving one party over another (e.g., passenger vs. pedestrian, or an old vs. a young pedestrian) in situations where harm is imminent and inescapable (e.g., Awad et al., 2018; Bonnefon et al., 2016). Most of this research has been modelled as a thought experiment based on the fabled *trolley dilemma* in moral psychology (Foot, 1967; Thomson, 1985). Specifically, an AV is proposed to be driving with passengers on board and some pedestrians suddenly appear in its path with no time to brake and completely stop. It has to decide between staying the course and running over the pedestrians or swerve to the side and hit a barrier that kills the passengers. How should the AV be programmed to decide about the appropriate action in this situation? What ethical rules or moral principles should it follow (protect the passengers at all costs or save the vulnerable pedestrians)? Finally, should the AV’s decisions be sensitive to specific characteristics of the potential targets of harm (e.g., their age, gender, social status, etc.)?

Apart from these trolley-type dilemmas, there are other ethical issues that have been highlighted for AVs (Himmelreich, 2018). For instance, the precise moment when AVs should yield to yellow traffic light signals, how close they should drive to bicycles, and should AVs cross over into other lanes to avoid obstacles (such as parked cars). Some researchers argue that these mundane ethical issues may be more relevant than the rare fatal circumstances entailed in the EDs, because they will be more frequent and thus are more pressing issues to resolve (De Frietas et al., 2020, 2021; Nyhoml & Smids, 2016; Himmelreich, 2018).

There are additional ethical issues pertaining to data privacy and potential for mass surveillance and hacking. AVs’ sensors collect tremendous amount of data about the location, traffic and behavior of various actors. While this data could help in navigation and monitoring of illegal activities, there is potential for mass surveillance of people and restriction on privacy (LaFrance, 2016). In addition, this data could land into the hands of bad actors and used for hacking and manipulating the operations of an AV (Lim & Taeihagh, 2018).

Among all the ethical issues, the EDs have garnered the most research and debate among academic scholars, AV manufacturers, and policy makers. This research and ongoing debates will now be discussed briefly.

## Research and debate on the relevance of EDs

The debate around EDs centers around two issues: (1) whether EDs are important at all for the adoption of AVs (and thus we should try to offer a solution to such dilemmas), and (2) is large scale public opinion data the best way to offer a solution to these EDs. The current work is focussed on the first issue and seeks to answer if EDs are indeed a critical issue to address for AV adoption. The second issue of whether public opinion is the best way to offer a solution to EDs, or it should be left in the hands of experts, is not the emphasis of the current research. This issue has been discussed extensively in other avenues (e.g., Rahwan, 2018; Awad et al., 2020a, b). As such, most behavioral studies on EDs have assumed that these dilemmas are important for consumers and thus appropriate solutions should be found. The next section briefly discusses these studies, followed by the criticism offered to this approach.

Several stylized versions of the classic trolley dilemma have been employed to examine the EDs posed for AVs. Pioneering work by Bonnefon et al. (2016) used these dilemmas to discover that while people expect AVs to be programmed with utilitarian ethics (e.g., sacrifice the passenger to save five or more pedestrians), they are reluctant to adopt such vehicles for their own use. Extending this to a one-to-one dilemma, Gill (2020) found that while people are pro-social as a driver in a regular car, they expect AVs to be more self-protective instead. The most comprehensive research along these lines is the moral machine experiment (MME; Awad et al., 2018). The MME used many variations of the ED and sought public opinion from millions of respondents from over 200 countries about how AVs should be programed to solve such a dilemma. The results from the MME revealed several strong preferences about AV ethics (e.g., protecting the young over old, and saving more over less) and some weak ones (e.g., protecting women over men, and the law-abiding citizens). Several cross-cultural variations in these ethical preferences also emerged. Overall, the MME has been touted as a significant and necessary public input for deciding upon the appropriate ethical programing of AVs, as not addressing this issue could lead to a public opt-out and rejection of such vehicles (Bonnefon et al., 2020a).

In contrast to the above, other academics believe that the ED is a distraction and of little practical value for the design of AVs (e.g., De Frietas et al., 2020, 2021). They argue that such dilemmas are incredibly rare and unlikely to occur, they cannot be easily detected by AV systems, and if detected they cannot be acted upon by a reliable control system (De Frietas et al., 2020). Similarly, Nyholm & Smids (2016) contend that while the ED is designed on outcomes with certainty, the real-life scenarios for AVs will be inherently probabilistic in nature and outcomes will be far from certain. Instead of a narrow focus on EDs, it is recommended that AVs should focus on the general principle of minimizing overall harm. Moreover, AVs should be designed for “common-sense driving” that can operate in more prevalent “low-stakes” ethical scenarios than the EDs (e.g., what to do if traffic light fails, whether to cross solid lines to avoid parked cars, etc.) (De Frietas et al., 2021; Himmelreich, 2018).

Some of the above arguments have indeed been rebutted by Wolkenstein (2018), who argued that EDs are not as rare as portrayed to be and that such dilemmas should be an inherent part of the overall moral competence designed into an AV. In particular, Bonnefon et al. (2019) contend that EDs are just a discrete limit case of the *statistical trolley problem*, wherein AVs will be continuously making decisions about distributing harm between passengers and other parties on the road. For instance, when deciding how close should AVs travel to bicyclists versus other moving vehicles, AVs will be making implicit decisions about distributing harm between the passengers on board versus other parties. The latter is in essence a low-stakes version of the ED.

Other stakeholders in this debate—namely, AV manufacturers and policy makers—also lean towards the view of moving away from designing specific solutions for EDs. Despite the widespread media attention, AV manufacturers have largely stayed away from such dilemmas and have allocated limited resources to designing appropriate solutions (Iagnemma, 2018; Olson, 2018). While one car manufacturer commented that their AVs will always prioritize the protection of their passengers from harm (Sorrel, 2016), this statement was quickly denied, and it was suggested that the company will implement whatever solution is found legally acceptable.

Among policy makers, various regulatory organizations in Europe and the U.S. are developing ethical guidelines for AVs and stipulating policies for their operation in the real world. These include the regulations and policies being discussed by the European Commission (CAV2020), Germany (Luetge, 2017), Canada (Canada DOT, 2019), U.S. (Administration NHTS, 2017), and Singapore (Council SS, 2019) to name a few. Some of these documents do specify policies pertaining to the ED. For example, the German ministry of transportation states that in the event of unavoidable accidents, the AV will not discriminate among individuals based on personal features such as age, gender, etc. This is counter to the findings of MME that people do want AVs to consider such features when deciding whom to save. Other documents, specifically, the European Commission’s CAV2020 recommends that AVs should follow the process of continuous risk management and focus on broader ethical principles (e.g., non-maleficence, beneficence, dignity, responsibility) in the design and operation of AVs.

While the debate about the importance of EDs for AVs continues, the opinion of one key stakeholder in this domain has been totally overlooked. Namely, the consumers or potential adopters of AVs, who will indeed determine the eventual success and tremendous benefits promised by this technology. Do consumers perceive EDs to be rare and thus less important as compared to the more prevalent and more likely technical or legal issues in the operation of AVs? There is a vast amount of literature on innovation adoption and on risk perception that can inform this issue, which is now discussed below. This is then applied to the context of AVs to speculate on the likely importance of EDs for the consumer adoption of such vehicles.

## Consumer perception of the benefits versus risks in innovation adoption

There is a large body of work in the management sciences on the process of innovation adoption, and the factors that hinder the consumer acceptance of new products (see Rogers, 1976, 1983 for the seminal works, and Hauser et al., 2006 and Arts et al., 2011 for a review of this literature). Among all the factors identified, the relative advantage or *benefits* (Rogers, 1983) and the *perceived risks* or costs (Ostlund, 1974; Ram & Sheth, 1989; Stone & Grønhaug, 1993) are the key factors that determine consumer acceptance versus resistance to innovations, respectively. This is especially the case for radical innovations that are significantly different from existing products and thus entail high uncertainty about the benefits as well as the risks in their adoption (Hoeffler, 2003). The risks in innovation adoption can originate from several sources including uncertainty about their performance, likely social and psychological consequences, switching costs, price, and potential for physical harm (Stone & Grønhaug, 1993). In addition, people show differential sensitivity to risks; wherein the mainstream consumers (about two-thirds of the market) are much more risk averse than the early adopters (Rogers, 1983). As such, a lot of innovations get traction with the early market but fail to be adopted by the mainstream consumers (Moore, 2002).

In the context of AVs, while the benefits of adoption are quite clear, in terms of lives saved and reduced congestion and health costs, the perceived risks are more in contention. Although Shariff et al. (2017) have explicitly pointed out that potential risks of negative consequences may be inflated for AVs, no research has examined the *relative* importance of the various risks associated with the different technical, legal and ethical issues discussed earlier. As such, there has been little theoretical discussion or empirical evidence on how consumers may weigh these different risks in the adoption of AVs.

Prior research has highlighted consumers’ differential sensitivity to the benefits versus risks when they considering adopting a new product (Gourville, 2003; Hoeffler, 2003). Consumers are loss averse and they weigh the risks in adopting new products much more heavily than the potential benefits of an innovation (Gourville, 2003). This follows from the extensive research on loss aversion in the decision sciences (Kahneman & Tversky, 1979). For instance, despite the significant benefits offered by electric cars (e.g., reduced pollution, lower cost of operation and lower maintenance needs) consumer adoption of these cars remains low (they account for less than 10% of the automobiles sold in the U.S.). Primarily due to the costs and risks associated with the lower travel distance for battery-operated cars, very long charging times and lack of enough charging stations. These costs, while objectively not so high, are subjectively weighed much more heavily than the benefits offered by electric cars (Gourville, 2003).

Similarly, for AV adoption decisions consumers are likely to weigh the risks or challenges more heavily than the benefits they may provide. Prior research has also shown that even if consumers state high intentions to adopt, they tend to get “cold feet” when the adoption time approaches (Alexander et al., 2008). This is because at the time for adoption consumers start paying more attention to the operational issues and risks associated with the innovative product as compared to its benefits. In sum, to predict AV adoption it is essential to understand how consumers would assess the risks associated with the different issues pertaining to the operation and usage of such vehicles.

The literature on risk perception has identified a strong reliance on feelings or affect in assessing risks; termed as the *affect heuristic* (Slovic et al., 2002). This heuristic suggests that people’s risk judgments rely heavily on the perceived feelings or emotions generated by an event rather than on cognitive deliberation and logical analysis about the properties of the event (Lowenstein et al., 2001; Slovic et al., 2002). This aligns with the rapid and intuitive feeling-based System 1 processing proposed in the dual process models of judgment and decision making (Kahneman, 2011). For instance, early research on risk perception showed that the feeling of dread determined the public perception and acceptance of hazardous technology, such as nuclear power plants (Fischhoff et al., 1978). This explains why public perceive a much higher risk from nuclear power plants than from medical x-rays, although this does not match the assessment by most risk experts (Fischhoff et al., 1978). Similar other research has shown that both risks and benefits of hazardous technology were judged based on perceived affect or feelings as compared to cognitive deliberations (Finucane et al., 2000).

Another characteristic of affect-based judgments of risks is their insensitivity to the probability of occurrence of an event; termed as *probability neglect* (Sunstein, 2003). When the consequences of an action or event carry strong affective reactions (positive or negative), such as for lottery winnings or plane crashes, their probability of occurrence carries little weight. Lowenstein et al. (2001) argue that for such events people’s risk assessments are highly sensitive to their strong positive or negative outcomes, regardless of their probabilities. Empirical evidence has confirmed this assertion. For instance, Rottenstreich and Hsee (2001) showed that when outcomes conveyed a strong positive or negative affect, people’s attractiveness or unattractiveness assessment was insensitive to probability variations as wide as from 0.01 to 0.99. This also explains the extreme overreaction of public officials and private citizens to potential terrorist threats, despite their much lower likelihood of occurrence compared to other daily hazards, such as road accidents (Sunstein, 2003).

The role of the affect heuristic and probability neglect in assessing the perceived risk associated with EDs, and its perceived importance to the adoption of AVs is discussed below.

## Consumer assessment of the perceived risk and importance of EDs for AV adoption

When viewed from the lens of the affect heuristic the ED entails a highly consequential outcome; namely, the possibility of fatal harm to the passengers. As such, this extremely negative outcome is likely to generate a very strong affective reaction among consumers. Even if the probability of occurrence of this dilemma may be rare, the strong negative feelings associated with the fatal outcomes would determine the risk perception. Just as people view plane crashes and terrorist attacks as highly risky, despite their very low probabilities, consumers are likely to view the EDs as a highly risky aspect of AVs. Furthermore, since the benefits and risks associated with a radically new product (such as an AV) are highly uncertain, as they are unfamiliar to consumers, any perceived risk will be viewed even more heavily than the same risk in a familiar product (Gourville, 2003; Hoeffler, 2003). This is evident from the tremendous media attention garnered by a single fatal accident with an AV as compared to the several more fatalities resulting from road accidents with regular vehicles (Knight, 2016).

Overall, the ED is associated with an extreme negative affect (due to potentially fatal consequences for customers), which would then lead to a very high risk associated with this dilemma. Furthermore, since the technology is new and highly unfamiliar, the risk is further exacerbated, and consumers will give significant importance to the need to overcome this ethical challenge for AV adoption. Note that there would indeed be a small section of consumers that may be more tolerant of these risks. But the majority of the potential adopters are likely to perceive a high risk in these EDs associated with the operation of AVs.

AVs also face numerous technical challenges as discussed earlier. For instance, inability to function well in chaotic inner-city environments (Gomes, 2014); susceptibility to bad weather conditions (Tussy, 2016); inaccuracy in recognizing all objects, road signs or unusual obstacles (Zhou, 2017); and inability to fully understand the gestures and non-verbal cues of other drivers and pedestrians (Gomes, 2014). These technical challenges are indeed important for the proper operation of AVs, and consumers will be cognizant of them when considering adoption. As such, most of these can be classified under the category of functional risks or performance uncertainty associated with new products (Stone & Grønhaug, 1993;﻿ Hoeffler, 2003). Many of these technical issues are indeed likely to be more frequently occurring issues than the EDs. However, these technical or performance risks would not be associated with a similarly strong negative affect as garnered by the EDs. Thus, despite their higher probability of occurrence, the risk perception and importance assigned by adopters to overcoming the above technical issues will be relatively lower than that assigned to the EDs.

One could argue that the above technical issues, such as the uncertainty about functioning in adverse weather conditions, could also lead to physical harm and fatal consequences for passengers. While this is indeed possible, it is contingent upon how easily such harmful consequences come to mind and how salient they are when consumers think about these technical issues for AVs. While for some consumers this may be salient, it is likely that most consumers do not directly associate or imagine physical harm due to these technical issues. For most consumers, potential for physical harm or fatality may be less salient than the operational/performance uncertainty evoked by these technical issues.

The legal issues about liability for harm caused by AVs are also being widely discussed. How much is the passenger versus the manufacturer or programmer liable for the damage caused by AVs and how should the current legal frameworks be adapted to accommodate these issues (Vladeck, 2014; Strong & Baker, 2015)? The liability issues can be considered a form of financial risk when consumers consider adopting AVs (Stone & Grønhaug, 1993). While financial risks are important in all adoption decisions, they do not entail the same aversive feelings that physical harm is likely to generate. Physical harm is more visceral and associated with strong emotions such as fear and dread, but financial harm is more cognitive rather than affective in nature. Thus, it can be expected that consumers will consider the legal challenges relatively less important to overcome than the ED.

Finally, apart from the EDs, there are other more “mundane” ethical issues that have been highlighted for AVs (Himmelreich, 2018). For instance, the precise moment when AVs should yield to yellow traffic light signals; and should AVs cross over into other lanes to avoid obstacles. There are also issues pertaining to data privacy and potential for mass surveillance and hacking (LaFrance, 2016; Lim & Taeihagh, 2018). All these issues are indeed important, but they do not entail the same extent of salient physical harm as EDs and thus will be associated with relatively lower risk.

The above discussion suggests that consumers considering the adoption of AVs are likely to weigh the ED as significantly riskier and a more important issue to overcome than the technical, legal or other ethical challenges. Two empirical studies were conducted to ascertain the validity of this proposition, which are now described in turn. All the material, survey instruments, and data from these two studies is available at: https://osf.io/buven/?view_only=281caf363f0441e19c0c88fd406bb1c8.

## Study 1: the relative importance of EDs for AV adoption

### Method

A survey was conducted with a quasi-representative sample of U.S. participants registered on the Prolific® research platform (*N* = 1,169*)* and were paid £1.00 each for their participation. ﻿Prolific®  is a crowdsourcing website whose participants are less familiar with common experimental paradigms and are deemed more honest than participants on MTurk, which is a more widely used participant platform (Peer et al., 2017). The study and all the materials were approved by the university research ethics board.

Participants were first presented detailed information about the benefits of AVs, and the different technical, legal/human and ethical challenges in the design and adoption of these vehicles. They then responded to comprehension questions pertaining to these benefits and each of the challenges. Respondents were not allowed to proceed further without correctly answering all these comprehension questions. This was to ensure that the respondents carefully read all the information and were aware of the benefits and the challenges for AVs. See the detailed study material and the survey instrument in the online supplementary information.

The respondents then rated the importance (on a 1(not at all)-to-7(very important) scale) of the different *benefits* (namely: reduced accidents, free-up commuting time, increase mobility for elderly and disabled, reduced traffic congestion, reduced insurance costs, and reduced health costs) provided by AVs. Subsequently, participants rated the importance of overcoming the different challenges—technical, legal/human and ethical—if they were to consider adopting an AV. The examined *technical* challenges were: inability to function in inner-city, susceptibility to weather, accurately recognize all objects, need for high-quality maps, changes needed in road infrastructure, read all road signs and symbols, and recognize gestures of pedestrians and other drivers. The *human*/*legal* challenges were: people enjoy driving and AV can take that away, laws and policy needs to be developed about who is liable for harm caused by AVs, unwillingness of people to give up control over driving, people employed in taxis and delivery services will lose jobs, and who pays for the fines when traffic rules are violated. Finally, the *ethical* challenges were: if harm is unavoidable should AVs harm the passenger or the pedestrian (the focal ED), should AVs violate traffic rules to avoid accidents, should AVs proceed through yellow light to avoid being rear-ended, if harm is unavoidable should AVs protect the young or old (an important demographic factor based on the MME), and the concerns about data privacy.

Following the above importance rating task, the participants then rated the *relative importance* of overcoming the different technical, legal and ethical challenges for AV adoption. Specifically, participants were asked to allocate 100 points among 10 different challenges (4 technical, 2 legal and 4 ethical) for the design and operation of AVs. This set of 10 included all the important challenges identified in the earlier importance ratings. Participants were informed that some of these challenges were less likely to occur then others, and they needed to use their own judgment to assess how important it is to overcome these challenges for them to adopt AVs.

In addition to the above 10 challenges, there was a dummy option that required respondents to fill in a zero in terms of points allocated. This was an *attention check* to make sure participants carefully followed the instructions for this crucial question about relative importance assigned to different challenges for AVs (163 participants failed this check and were excluded, resulting in the final sample size of *N* = 1,006; see Table 1 for the detailed sample characteristics).

**Table 1:** Sample characteristics for study 1 (*N* = 1,006)

Measure	Mean/Frequency
Age	M (SD) = 44.14 (16.73) years; 18–80 years
Gender	46% male; 53% female; 1% other
Have children	50% yes; 49% no; 1% didn’t say
Ethnicity	69% white; 11% black; rest others
Education (highest level)	11% high school; 31% college or associate degree; 31% bachelor’s; 27% graduate degree
Income	20% < 25 k; 24% 25–49 k; 20% 50–74 k; 15% 75–100 k; 21% > 100 k
Driver’s license	92% yes; 8% no
Driving experience	6% < 1 year; 6% 1–2 years; 6% 3–4 years; 8% 5–6 years; 74% > 6 years
Usually passenger or driver in car	19% passenger; 54% driver; 27% both equally
Prior exposure to AV dilemmas	85.5% no; 14.5% yes
Prior exposure to AV studies (1–9 scale)	M (SD) = 2.54 (1.96)

Respondents then evaluated the AV on *attitudinal* measures (how excited, fearful they were of AVs; both 1(not at all) to 9 (extremely) scales) and on their *adoption intentions* (how likely are they to purchase an AV if it is available and they are about to purchase a new car; on a 1(not at all) to 9 (extremely likely) scale). Subsequently they provided background information about their *prior exposure* to research studies on AV dilemmas (have they participated in AV dilemma studies (yes/no)); how much exposure they had to research studies on AVs (1(not at all) to 9 (a lot)); do they have a driving license (yes/no); their *driving experience* (from less than a year to more than 6 years); and their status in a car (are they usually the passenger, driver, equally both or do not ride). Finally, the respondents provided demographic information (age, gender, have children, ethnic background, education, and income) and their political orientation (two 7-point scales anchored by liberal vs. conservative and democrat vs. republican). As a follow-up, further questions elicited the respondents’ own ethical preferences about how AVs should be programmed to resolve the focal ED (of protecting the passenger vs. pedestrian). These are described later in the General Discussion section.

### Analysis and results

#### Importance ratings

**Fig. 1:**
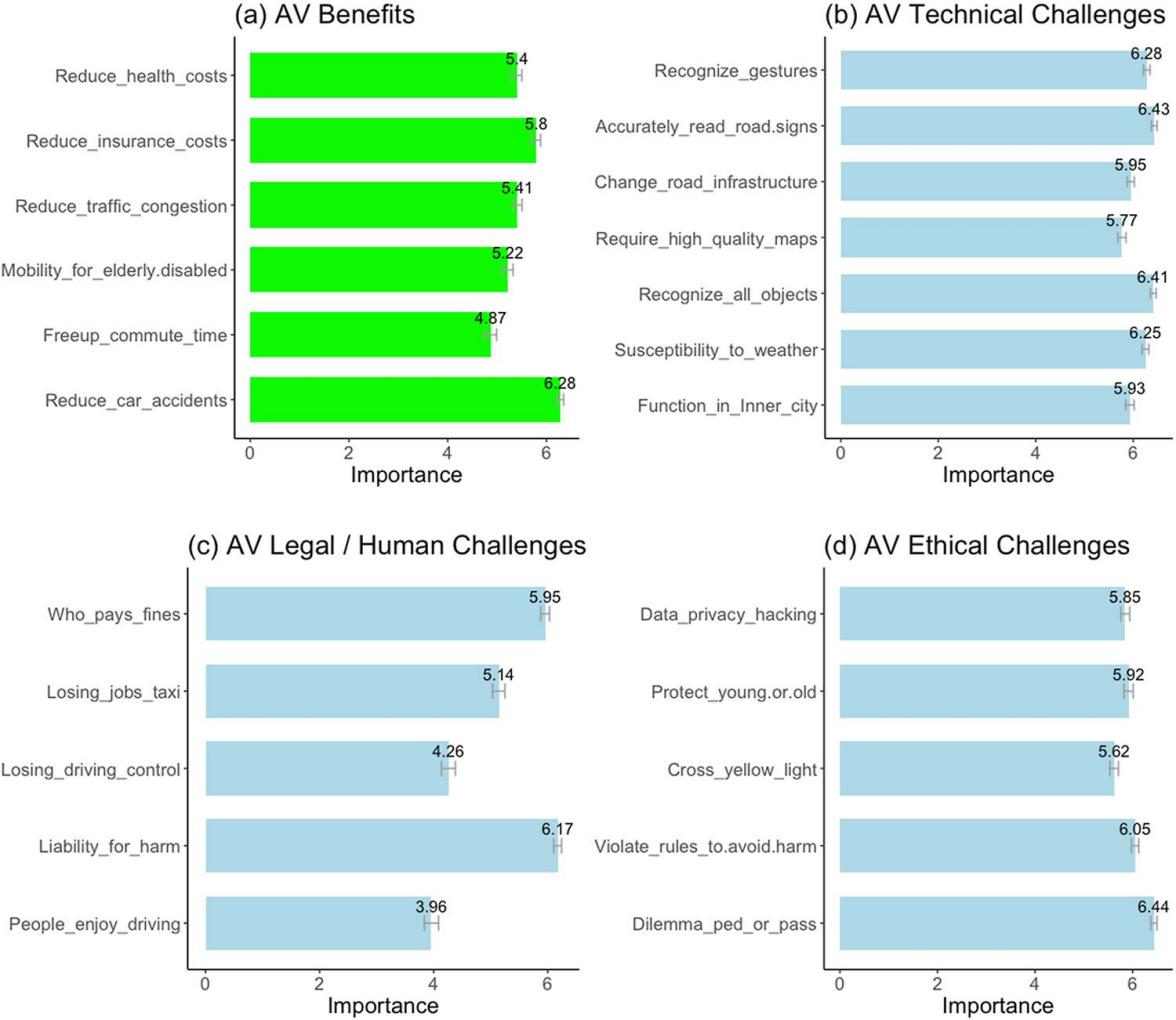
Importance ratings of the benefits and challenges in the adoption of AVs (study 1). Participants (*N* = 1,006) rated how important were the listed benefits and how important it was to overcome the listed technical, legal / human, and ethical challenges if they were to consider adopting an AV. All ratings were on 1(not at all)-to-7(very important) scales

The descriptive results from the survey for the importance ratings of the benefits of AVs and the importance of overcoming the different challenges are shown in Fig. 1. Among the benefits, people rated reduced car accidents as the most important benefit of AVs followed by reduced insurance costs and reduced traffic congestion. Among the technical challenges the significantly important ones (rated higher than 6 on the 7-points scale) were the ability to accurately read road signs, recognize all objects, recognize gestures, and susceptibility to weather. For the legal challenges the liability of harm was the most important. Among the ethical challenges, the most important one (rated 6.44 on the 7-point scale) was the focal ED of whether to harm pedestrian or passenger if harm is unavoidable (see Fig. 1 for details).

#### Relative importance

**Fig. 2:**
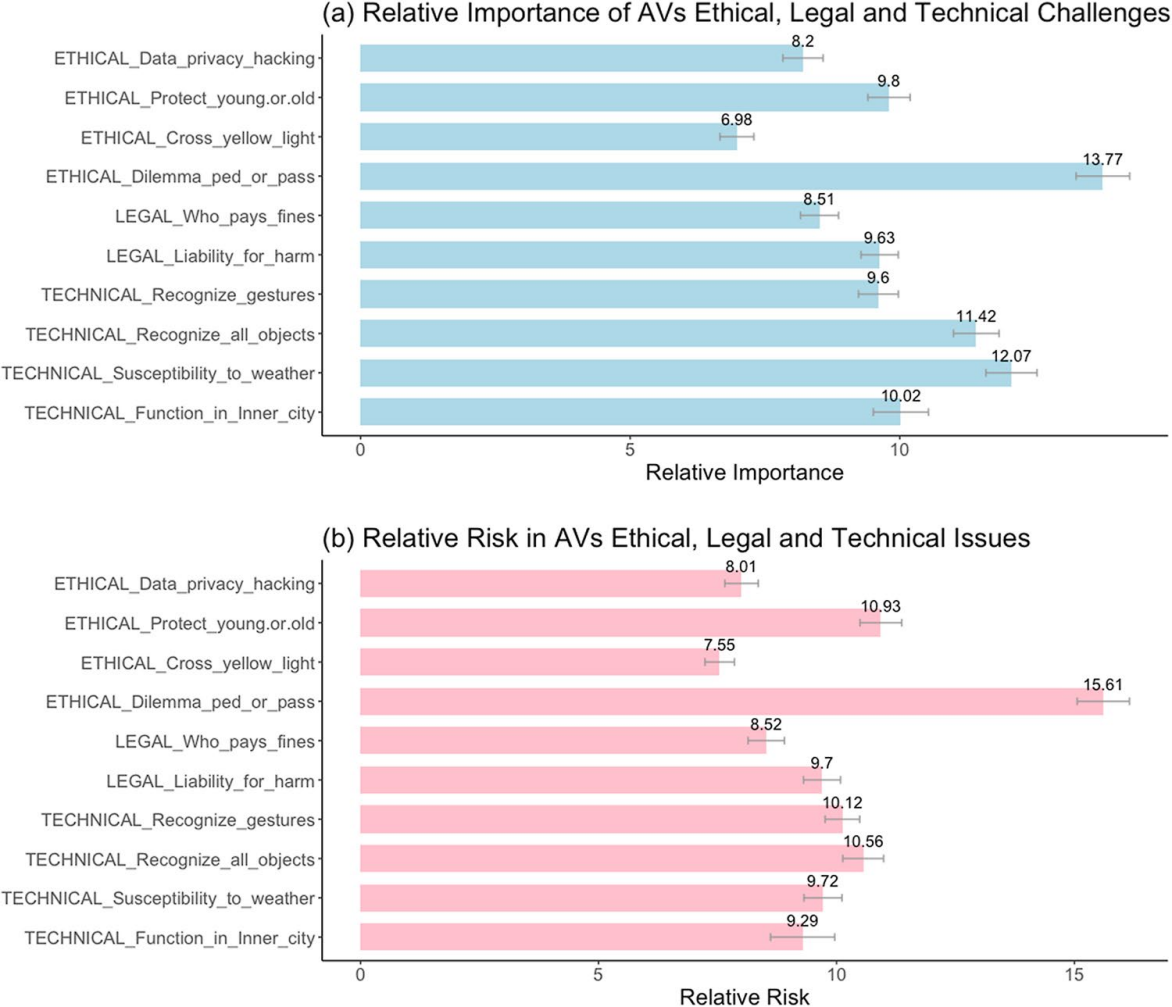
**a** Relative importance of the challenges in the design and operation of AVs (study 1). Participants (*N* = 1,006) were asked to allocate 100 points among the 10 listed ethical, legal and technical issues based on their perceived importance to overcome these challenges for adoption of AVs. **b** Relative risk of the different issues in the design and operation of AVs (study 2). Participants (*N* = 672) were asked to allocate 100 points among the 10 listed ethical, legal and technical issues based on how much risk they perceived in these issues for AVs

The relative importance of the above challenges revealed an insightful picture, as shown in Fig. 2a. Among the ten selected technical, legal and ethical challenges (which included all the ones rated as highly important), the focal ED for AVs was allocated with the highest relative importance rating (*M* = 13.77; *SD* = 8.03). Table 2 shows the mean relative importance ratings for these ten challenges and Table 3 shows a statistical comparison across these ratings. As evident from Table 3, the focal ED was rated as significantly more important than all the other challenges (all *p*s < 0.001 for all the nine pairwise comparisons). Further analysis revealed that the above result was not influenced by the participant’s prior exposure to AV dilemmas (see fig. S1a in the accompanying supplementary information). The demographic factors also did not affect these results.

**Table 2:** Relative importance of AV challenges: ethical, legal and technical (study 1)

Measure	N	Mean	SD
Importance_Ethical_dilemma_pass_ped	1006	13.766	8.035
Importance_Tech_innercity	1006	10.021	8.262
Importance_Tech_weather	1006	12.071	7.659
Importance_Tech_recognize_objects	1006	11.418	6.839
Importance_Tech_recognize_gestures	1006	9.604	5.967
Importance_Legal_liability_harm	1006	9.626	5.583
Importance_Legal_who_pays_fines	1006	8.511	5.710
Importance_Ethical_yellow_light	1006	6.979	5.082
Importance_Ethical_data_privacy	1006	8.204	6.025
Importance_Ethical_protect_young_old	1006	9.800	6.336

**Table 3:** Relative importance of AV ethical dilemma versus other challenges (study 1)

Measure 1	Measure 2	t	df	p	Cohen’s d
Importance_Ethical_dilemma_pass_ped	Importance_Tech_innercity	9.018	1005	< .001	0.284
Importance_Ethical_dilemma_pass_ped	Importance_Tech_weather	4.292	1005	< .001	0.135
Importance_Ethical_dilemma_pass_ped	Importance_Tech_recognize_objects	6.248	1005	< .001	0.197
Importance_Ethical_dilemma_pass_ped	Importance_Tech_recognize_gestures	11.913	1005	< .001	0.376
Importance_Ethical_dilemma_pass_ped	Importance_Legal_liability_harm	12.778	1005	< .001	0.403
Importance_Ethical_dilemma_pass_ped	Importance_Legal_who_pays_fines	15.563	1005	< .001	0.491
Importance_Ethical_dilemma_pass_ped	Importance_Ethical_yellow_light	22.162	1005	< .001	0.699
Importance_Ethical_dilemma_pass_ped	Importance_Ethical_data_privacy	17.230	1005	< .001	0.543
Importance_Ethical_dilemma_pass_ped	Importance_Ethical_protect_young_old	14.934	1005	< .001	0.471

Student’s t-test based on pair-wise mean comparisons

#### Attitudes and purchase intentions

The attitudes towards AVs were ambivalent. Participants reported both excitement (*M* = 5.38; *SD* = 2.61) and fear (*M* = 5.51; *SD* = 2.29) towards these vehicles (both 9-point scales). This is consistent with prior research on hazardous technology, such as nuclear power plants, where both benefits and risks were equally prevalent (Finucane et al., 2000). The purchase intentions for AVs were relatively low (*M* = 4.10; *SD* = 2.65; on a 9-point scale). The reported purchase intentions for AVs was regressed on the perceived benefits, relative importance of the ten challenges for AVs, and the demographics and other measures. A stepwise regression was conducted to ascertain which of the above predictors were significant and improved the fit of the model. The final regression model (to which adding more variables did not improve fit) included four of the AV benefits, some control measures, and only one of the ten challenges. Specifically, only the ED’s relative importance ratings entered as a significant negative predictor for purchase intentions. None of the technical, legal or other ethical challenges improved the fit of the model. See online supplementary information for details.

## Study 2: the relative risk associated with EDs for AV adoption

Another study was conducted to further verify the above results about the relative importance of EDs for consumer adoption of AVs. In this study, participants directly indicated the risk associated with the different technical, legal and ethical issues examined earlier. This was to ascertain whether people indeed perceived significant risks with each of these issues and framing them explicitly as risks versus challenges does not alter the previous findings.

This study was conducted with a different sample that is still relevant to AV adoption. Namely, undergraduate business students enrolled in a Canadian university participated for extra course credit. Participants (*N* = 672 after excluding 51 that failed the attention check; 54% males; mean (SD) age = 19.7 (1.27) years; 95% had a driving license; 87% no prior exposure to AV dilemma; ethnicity = 50% white, 21% south Asian; 18% east Asian; and 11% others) responded to the same survey instrument as used in the earlier study. The only difference was that participants explicitly rated the *perceived risks* (on 1(no risk at all) to 7 (very high risk) scales) associated with each of the technical, legal and ethical issues examined before. Also, *relative risk* was measured by asking participants to distribute 100 points based on the perceived risks in the same set of ten issues examined earlier. All other measures were the same as before.

The results for perceived risks were similar to those obtained for the importance ratings in the earlier study (see supplementary information). More importantly, the relative risks, as shown in Fig. 2b, mirrored the results obtained in study 1. As before, the focal ED for AVs was associated with the most risk, which was significantly higher than the risk perception for each of the other technical, legal or ethical issues. Also, these relative risks were not affected by prior exposure to such AV dilemmas (see fig. S1b in the supplementary information). As such, these results replicate and confirm the earlier results from study 1.

Overall, the results from the two studies show that the central ED for AVs—protecting / harming the passenger or the pedestrian—is associated with significant risks and is perceived as a critically important challenge to overcome for the adoption of AVs. Crucially, this trolley-type dilemma was perceived by potential adopters as significantly riskier and a more important challenge to overcome than any of the key technical, legal or other ethical challenges examined for AVs.

## General discussion

The ethical challenges facing AVs—especially the trolley-type dilemmas of protecting the passengers versus pedestrians—has been a topic of intense investigation as well as of debate. The highly publicized MME (Awad et al., 2018) and other prominent works (Rahwan, 2018; Awad et al., 2020a, b) are based on the premise that EDs are a critical challenge to address for the consumer adoption of AVs. Not adequately addressing this issue could lead consumers to shun AVs, and thus nullify all their promised benefits (Bonnefon et al., 2020a). But several academics (Nyhoml & Smids, 2016; Dewitt et al., 2019; De Frietas et al., 2020, 2021) and AV manufacturers (Iagnemma, 2018; Olson, 2018) have questioned the importance of such investigations and claimed that EDs are a diversion as compared to the other more significant technical challenges in the proper functioning of AVs.

However, conspicuously absent from this debate is the opinion of one of the most important participants in the success of AVs; namely the consumers or potential adopters of such vehicles. The current research investigated this issue both from a theoretical standpoint and through empirical research. Two studies were conducted with a broad sample of consumers about the relative importance and risks they placed on the ED versus the technical, legal and the other ethical issues in the adoption of AVs. The results from these studies suggest that in the eyes of adopters, EDs are the most critical issue to resolve to enable the adoption of AVs.

The extensive literature on innovation adoption identifies perceived benefits versus risks as the key factors in consumer’s receptivity versus resistance, respectively, to radical innovations (Ostlund, 1974; Rogers, 1983; Ram & Sheth, 1989; Stone & Grønhaug, 1993). The risk perceptions are driven by the strength of the affect associated with the potential negative consequences of adoption (Slovic et al., 2002). Moreover, these risk assessments virtually neglect the probability of occurrence of adverse events and primarily focus on the severity of their consequences (Lowenstein et al., 2001; Rottenstreich & Hsee, 2001, Sunstein, 2003). This prior research suggests that the EDs would be perceived as highly risky as they entail a potential for fatal outcomes for AV adopters. As such, these aversive outcomes are likely to garner a stronger negative affect than the harm resulting from the other technical issues or legal concerns. Thus, based on the extant literature, the importance of resolving the ED is likely to be weighted more heavily in consumers’ minds than the technical or legal issues in the design of AVs.

Two empirical studies verified the above assertion. In study 1 conducted with a broad sample of the US population it was found that the ED of protecting the passengers versus pedestrians in case of unavoidable harm was considered the most important challenge to overcome. Study 2 further showed that the explicit risks associated with this ED were significantly higher than the risks perceived for the main technical or legal issues. Moreover, the high relative importance and risk accorded to EDs was not influenced by prior exposure to such dilemmas.

Shying away from EDs or ignoring their importance due to their low probability of occurrence would be akin to focussing on the “normal” and ignoring such rare events that could have a huge downside, as highlighted in the opening quote. While EDs are not a Black Swan event (an event that is inherently unpredictable and unknowable; Taleb, 2007) they can pose a large “tail risk” (Cirillo & Taleb, 2020). EDs can be considered low probability events that are foreseeable and carry very large downside risks, but their precise time of occurrence is not predictable (e.g., the recent Covid-19 pandemic; Taleb, 2007). Most of the decisions made by AVs during their “normal” operation will garner very little perceived risks for adopters (e.g., keeping in the center of the lane, following traffic rules, etc.). But there will be some decisions that while being extremely rare will have a huge downside risk. EDs can be considered one such “edge case” wherein the AV will need to prioritize between harming passengers versus pedestrians or cyclists. Note that while this edge case of EDs with potentially fatal outcomes is likely to be rare, there will be several low-stakes versions of these EDs that will be more frequent (e.g., how much space to leave when passing a bicyclist on a two-way traffic lane).

The downside tail-risk of EDs is not primarily in the fatalities that could be caused by such events, but in the large negative effect that even a single ED like event can have on public trust in AVs. For instance, when Uber’s self-driving car failed to detect and killed a pedestrian in Arizona in 2018 it stoked public fear of AVs, and Uber decided to shelve its AV program entirely (Topham, 2021). While any fatality involving an AV will raise fear and doubt, the negative public reaction following an ED will be further amplified by the accompanying moral outrage. One can imagine that rare events such as EDs can and will occur once AVs have spent enough time on the road. If an ED results in a fatality—whether it is a passenger or a pedestrian—there would be immense scrutiny about the ethical calculus governing AVs. This will likely have a ripple effect on consumer confidence, and significantly reduce trust in this technology.

Analyst believe that after their peak hype in 2015, the AV industry has come to realize that getting the technology to work perfectly in real-world conditions is a much bigger challenge than anticipated (Topham, 2021). Consumers do expect autonomous technology to be perfectly safe and are extremely unforgiving of any errors made by such systems, as shown by the growing body of research on “algorithm aversion” (Dietvorst et al., 2015﻿; Burton et al., 2020). Recent research has shown that people expect extreme safety from AVs, and they will consider adoption only when AVs prove to be substantially safer than human drivers (Shariff et al., 2021). The current findings show that among all the safety issues, people perceive the challenge in addressing EDs as the most significant concern in the adoption of AVs.

Addressing EDs is indeed a non-trivial task for designing and regulating AVs. Several scholars and manufacturers point out the near impossibility and frivolousness of solving this issue (De Frietas et al., 2020; 2021). On the other hand, behavioral science researchers consider public opinion as a necessary input in deciding about the ethical principles for AVs (Awad et al., 2018, 2020a, b). Many policy makers, such as the European Commission’s CAV2020 report have suggested that EDs can be adequately solved if AVs follow a continuous risk management process in its operation. The report suggests that “the behaviour of a CAV in a dilemma situation is by default acceptable if the CAV has, during the full sequence that led to the crash, complied with all the major ethical and legal principles stated in this report” (p. 33; CAV2020). In other words, AVs should focus on operational safety and continuously manage and distribute risks across different parties (e.g., passengers versus cyclists or pedestrians), which will allow for a fair distribution of residual risk if an ED occurs.

While reasonable in principle, these guidelines may not be enough to assuage consumers’ fear, uncertainty, and doubt about AVs. Since consumers will be ceding control of all the driving operations to an AV, they will want that the AV considers their own protection as paramount. One can imagine that people who take an AV for a test drive will be observing the behavior of these vehicles and assessing how safe they feel riding in them. From what they observe, people will implicitly assess how much priority the AV is giving to the safety of the passengers on board versus others on the road. For example, if an AV leaves a lot of space when passing a bicyclist and veers too much into the incoming traffic lane, people on board will be scared and feel unsafe. How AVs react to such ED-like scenarios will be a key aspect of how adopters assess the risks in riding such vehicles. Consumers need assurance that AVs will protect them and their families riding in the vehicle if ever an ED-like event arises. The rarity of this event may not be enough of an assurance to majority of adopters, as evident from the two studies reported earlier.

Given the reported critical importance of EDs, future research should identify ways to address these dilemmas. This may be especially important in the early stages of AV adoption, wherein the ethical preferences of early adopters need to be considered to gain initial acceptance. Furthermore, the social dilemma in the consumer preferences for the ethics of an AV (Bonnefon et al., 2016) would also need to be addressed. The next section discusses some preliminary findings about addressing these issues and suggests avenues for future research.

### How to resolve EDs for the likely early adopters of AVs

Addressing EDs will require collaboration and expertise from the domains of ethics, philosophy, government policy, law, computer science and automotive engineering. However, as advocated in the society-in-the-loop framework (Rahwan, 2018), seeking public opinion on this matter can provide guidance to addressing AV ethics. The MME and several other studies have already provided evidence about the consumer preferences for how AVs should resolve EDs. While this evidence is very useful at the aggregate level for the population as a whole, it does not directly resolve the issue that is going to be imminent for individual adopters of AVs. Namely, when people adopt AVs for their own use their ethical preferences are likely to be different and more self-serving than what they advocate for AVs in general (as revealed in the *social dilemma﻿* of AV ethics; Bonnefon et al., 2016). To aid adoption, it will be important to find ways to resolve this social dilemma so that the ethical preferences of individual adopters are aligned with that of the society as a whole.

As a follow-up to previous studies, some preliminary evidence was collected about the effectiveness of using different behavioral science-based interventions to resolve the social dilemma in AV ethics. In addition, the ethical preferences of likely early adopters of AVs was also ascertained. The same set of participants as in studies 1 and 2 responded to follow-up questions about their ethical preferences for how AVs should resolve the ED. Specifically, the participants were randomly assigned to one of six conditions where they were asked if an AV faced a situation of unavoidable harm should it be programmed to protect the passengers or the pedestrians or have no priority.^2^ The different conditions were created by manipulating the framing of the question about the ethical preferences.

In one condition the question was framed as “Which of the three ethical preferences—‘protect passengers over pedestrians’, ‘protect pedestrians over passengers’, or ‘no priority’—should be programmed into an AV” (termed as the *others* condition). In the second condition, the question mentioned that the AV was to be purchased by participants for their own use (*self-buy* condition), and in the third condition it was stated that the AV was to be rented for their own use (*self-rent* condition). The fourth condition said the AV was purchased for own use and employed the behavioral intervention of social proof or descriptive social norms (i.e., influence of the majority; Cialdini, 2001). Specifically, it stated that “in a similar previous research most people chose AVs to be programmed to protect the pedestrians or to have no priority” (*self-buy-norm* condition). The fifth condition used the intervention of defaults (Johnson & Goldstein, 2003) and stated that the AVs were programmed to have “no-priority” as default, but the respondents could change this for their own AV (*self-buy-default* condition). Finally, the sixth condition used the intervention of virtue signalling (Griskevicius et al., 2010), stating that the participants’ chosen ethical preference for their AV will be publicly visible to others (*self-buy-visib* condition; this intervention was suggested in Shariff et al., 2017). See the supplementary information for details.

**Fig. 3:**
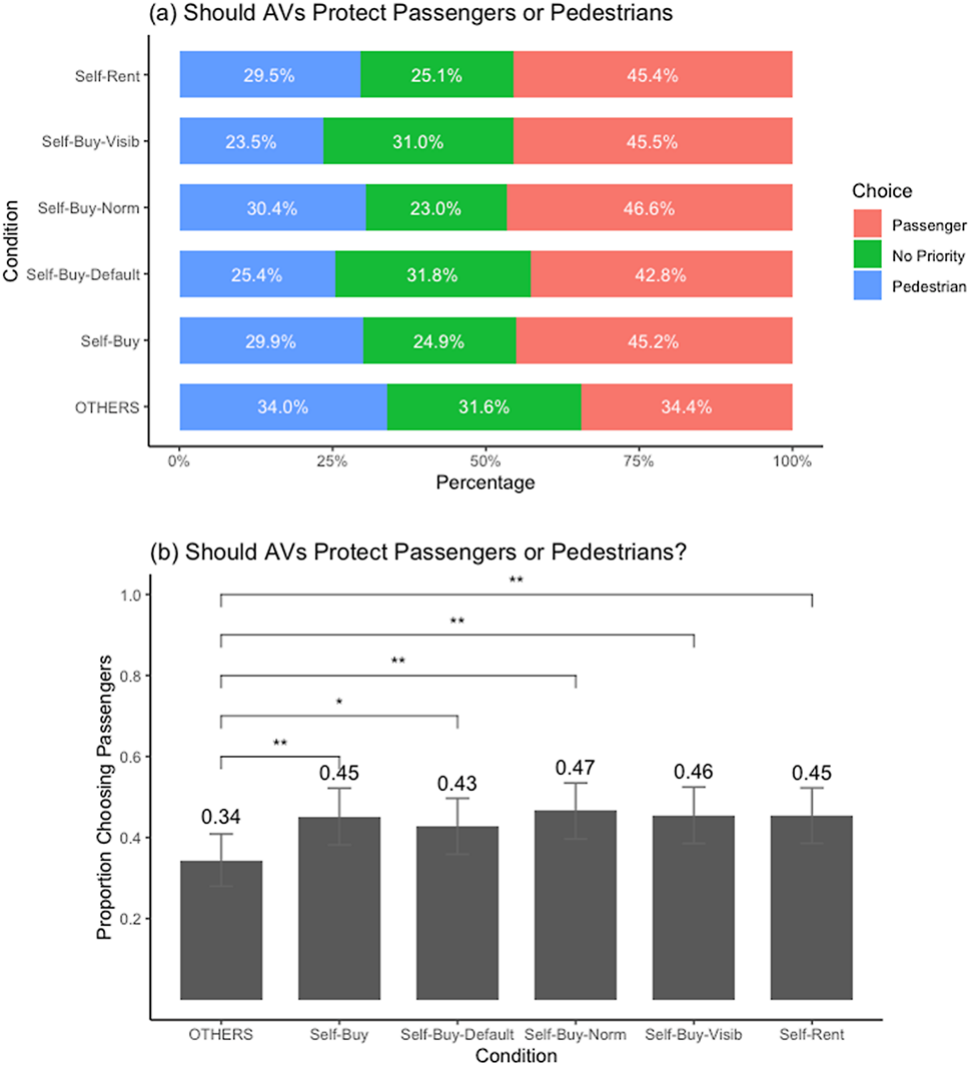
Ethical preferences for AVs (study 1 follow-up). **a** Participants were asked how should AVs be programmed in a dilemma where harm was unavoidable: “protect passengers over pedestrians”, “protect pedestrians over passengers”, or “no priority”. They were randomly assigned to one of six conditions: (1) AVs in general (*others*), (2) AVs bought for own use (*self-buy*), (3) AVs for own use with default setting of no priority (*self-buy-default*), (4) AVs for own use stating that majority prefer protecting passengers or no priority (*self-buy-norm*), (5) AVs for own use with visible ethical settings (*self-buy-visib*), and (6) AVs rented for own use (*self-rent*). **b** Pairwise comparisons with a student-t test (*p < .10; **p < .05; ***p < .01; ****p < .001)

The results from the above follow-up questions are shown in Fig. 3. These results revealed that, as shown in previous research (Bonnefon et al., 2016), there was indeed a social dilemma in the consumer preferences for AV ethics. That is, compared to the condition about general preferences for AVs (*others* condition), a significantly higher proportion of respondents chose the option of “protect passengers over pedestrians” in all the other five conditions that entailed AVs for their own use. However, the ethical preferences in the latter five conditions did not differ from each other. In other words, none of the tested interventions worked to eliminate the social dilemma in AVs ethical preferences.^3^ Note that these were descriptive scenarios that did not entail any real consequences for participants. Future research should examine these interventions further using more consequential scenarios or test other potential interventions to resolve the social dilemma in AV ethics.

**Fig. 4:**
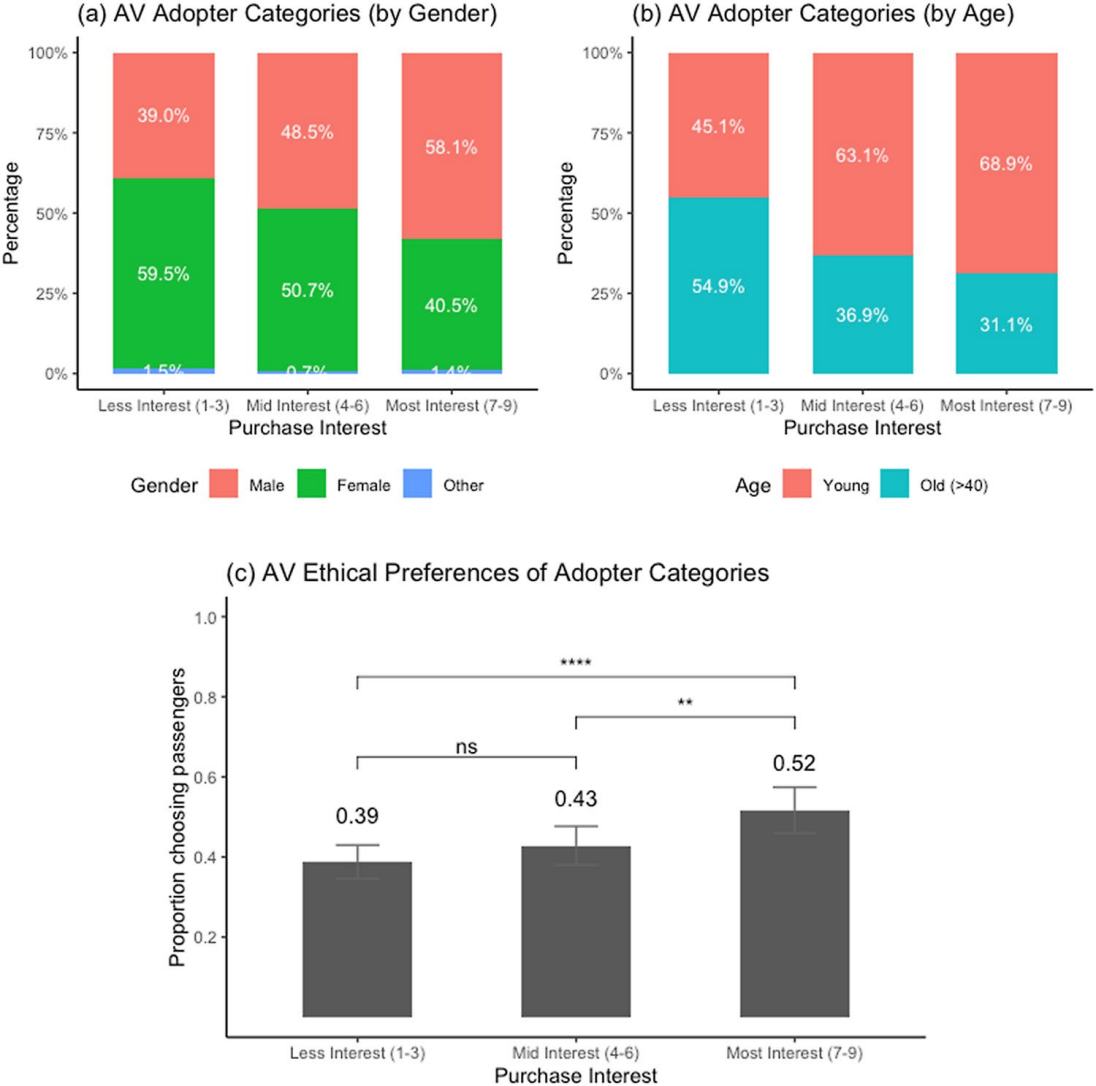
AV adopter categories based on purchase interest (study 1 follow-up). **a** Category of adopters based on reported purchase interest in AVs. The group with the most purchase interest (7–9) are deemed as likely *early adopters*, and they have a relatively higher proportion of males than the other two groups. **b** The likely early adopters group have a relatively higher proportion of young (< or = 40 years old) people than the other two groups. **c** The prospective early adopters are more self-serving than the other groups (i.e., a higher proportion choose the option of “protecting passengers over pedestrians” in their ethical preferences for AVs). Pairwise comparisons with a student-t test (*p < .10; **p < .05; ***p < .01; ****p < .001)

Further analysis was conducted to ascertain the ethical preferences of likely *early adopters* of AVs. Participants were classified into three categories based on their reported purchase interests for AVs: “less interest” (scoring between 1 to 3 on the 9-point purchase interest scale; 43% of the sample), “mid interest” (scoring 4 to 6; 33% of the sample), and “most interest” (scoring 7 to 9 on the purchase interest scale; 24% of the sample). As shown in Fig. 4, the latter group with the most purchase interest—deemed as likely early adopters—constituted a relatively higher proportion of males and was of a younger age profile (< or = 40 years old) as compared to the other two groups. The ethical preferences of these three groups (shown in Fig. 4) revealed that the early adopters were significantly more likely to show a self-serving bias in AV ethics (i.e., they were more likely to choose the “protect passengers over pedestrians” option than the other two groups). In addition, the ethical preferences of early adopters also did not exhibit a social dilemma in their preferences. That is, their preferences for AVs designed for others versus for self were aligned and more self-serving than those of the other groups. See supplementary information for details.

Overall, this further analysis revealed that early adopters of AVs are likely to be younger males and they would prefer AVs that protect themselves over pedestrians in an ED where harm is unavoidable. Future research should probe further into these ethical preferences of the prospective early adopters. Testing these ethical preferences in low stakes ED-like scenarios would be particularly useful to gauge the perceived risks and safety in riding AVs.

## Conclusions

In conclusion, while EDs are edge cases they carry a severe downside risk that can stall the adoption of AVs. Critics claim that EDs are rare and undetectable, and thus a distraction for the design of AVs. EDs with fatal outcomes may be rare, but low-stakes ED-like scenarios will be quite common, and potential adopters will use these to assess the risks in riding an AV. The need to find an acceptable solution to this issue is to ensure robustness in the design of AVs, and to assure consumers of the safety of these vehicles. While the other operational-level technical issues remain critically important for the success of AVs, addressing EDs seems to be the most important challenge in the eyes of adopters. A viable solution to EDs would be crucial to get traction among the early adopters of AVs, who will set the stage for the wider diffusion of this promising technology.

## Supplementary Information

Below is the link to the electronic supplementary material.

Supplementary file1 (DOCX 155122 kb)

## Data Availability

The datasets generated during and/or analysed during the current study are available in the OSF repository, here: https://osf.io/buven/?view_only=281caf363f0441e19c0c88fd406bb1c8.

## Footnotes

1 Adopters here are referred to as individual consumers who will be the end users of AVs, whether by purchasing one solely for their own use or by hiring, renting or sharing one on a pay-per-use basis.

2 A recent study (Bigman & Gray 2020) challenged the MME and suggested that participants in these trolley-type dilemmas were presented with a forced dichotomous choice between protecting passengers versus pedestrians (or young vs. old, etc.). In multiple studies, Bigman and Gray (2020) showed that people overwhelmingly preferred the choice of “equal treatment” of targets (passengers vs. pedestrians) if this option was available. In light of this, in the follow-up questions to study 1, participants were presented with the third option of AV programmed with “no priority” to protect passengers versus pedestrians if harm was unavoidable. Similarly, in the follow up to study 2 the third option was worded as “equal priority” to protect passengers and pedestrians. The study 1 follow-up included some additional participants to achieve sufficient power in the analysis of the different interventions.

3 A similar analysis was conducted on the follow-up ethical preference questions in study 2. The results were qualitatively similar to those obtained here, except that the third option of “equal priority” (to passengers and pedestrians) was chosen more often than the “no priority” third option used in the study 1 follow-up. That said, the social dilemma still persisted in the ethical preferences for AVs; but it was less pronounced across conditions.

## Code availability

Available upon request from the author.
