# Supplementary material for: Ethical dilemmas are really important to potential adopters of autonomous vehicles
Source: Ethics Inf Technol. 2021 Jul 2;23(4):657–73. doi: 10.1007/s10676-021-09605-y (PMC8253245; doi:10.1007/s10676-021-09605-y)
Supplement: Supplementary file 1 — Supplementary file1 (DOCX 155122 kb) [file 10676_2021_9605_MOESM1_ESM.docx]

**SUPPLEMENTARY INFORMATION**

**Ethical Dilemmas are Really Important to Potential Adopters of Autonomous Vehicles**

This file includes:

**Supplementary Methods**: Survey Instrument for Study 1 and follow-up questions.

**Supplementary Tables S1 to S3**: Detailed analysis & results for studies 1 and 2.

**Supplementary Figures S1 to S3**: More results from studies 1 and 2.

**Supplementary Methods**

**Survey Instrument (Study 1)**

**CARS STUDY - 2020**

This study seeks your opinion about certain type of cars; namely, **self-driving cars.**

The data collected in the study will be strictly kept confidential.

There are no right or wrong answers to any of the questions, so please be as honest as possible in your responses.

**It is important that you take the study seriously and pay attention to the questions asked, if you don't, it can invalidate the responses and the data collection will not serve the purpose.**

Are you ready to participate in the study?

Click below to start.

**SELF-DRIVING CARS**

**Please read the information below very carefully and answer the questions that follow.**

**--> There will be attention checks based on your understanding of the information below.**

**Self-Driving Cars**

A **self-driving car**, also known as an **autonomous vehicle** (**AV**), **driverless car**, **robocar**, or **robotic car** is a vehicle that is capable of sensing its environment and moving safely with little or no human input. Most of the leading automakers in the world (e.g., Ford, BMW, GM, etc.) and tech companies (Google, Telsa, Uber) are investing heavily in self-driving cars and expect to soon introduce them to the market.

**BENEFITS OF SELF-DRIVING CARS**

- Every year close to 1.25 million people die worldwide of car accidents and 35,000 people die in the U.S. of car accidents. According to National Motor Vehicles Crash data more than 94% of accidents are caused by human driver error, such as delayed reaction time, tailgating, rubbernecking, and other forms of distracted or aggressive driving. Adoption of self-driving cars can reduce / eliminate these errors and an estimated 25,000 lives would be saved annually in the U.S.
- Self-driving cars could relieve travelers from driving and navigation chores, thereby replacing behind-the-wheel commuting hours with more time for leisure or work.
- For the young, the elderly, people with disabilities, automated cars could provide enhanced mobility.
- Additional advantages include higher speed limits; smoother rides; and increased roadway capacity; and minimized traffic congestion, due to decreased need for safety gaps and higher speeds
- Self-driving cars and safer driving is expected to reduce the costs of vehicle insurance.
- Lives saved by averting automobile crashes in the US can save health costs of more than $200 bil.

**TECHNICAL CHALLENGES for SELF-DRIVING CARS**

- Artificial Intelligence is still not able to function properly in chaotic inner-city environments.
- Susceptibility of the car's sensing and navigation systems to different weather (such as rain, snow).
- Inability to recognize all objects and obstacles, and accurately read all traffic signs and signals.
- Autonomous cars require high-quality specialised maps to operate properly, not widely available.
- Current road infrastructure may need changes for automated cars to function optimally.
- Inability to read the non-verbal signs and gestures from pedestrians and / or other drivers.

**HUMAN & LEGAL CHALLENGES for SELF-DRIVING CARS**

- Reluctance by individuals to give up control over driving.
- Some people enjoy driving and self-driving cars could take that away.
- People may lose jobs who rely on driving as a means of work (e.g., taxis, delivery, etc.).
- Laws and policy are being developed about who is liable when an automated car causes physical damage to persons and / or breaks road rules.
- Who is responsible for damage and injury and need to address the potential for conflicts of interest between human occupants, system developers, manufacturers, insurers, and the public purse.\

**ETHICAL CHALLENGES for SELF-DRIVING CARS**

- How automated vehicles should be programmed to behave in an emergency situation where either passengers or other traffic participants like pedestrians, bicyclists and other drivers are endangered.
- If harm is unavoidable, how should a self-driving car decide between harming / killing pedestrian(s) versus harming / killing the passenger(s).
- Should self-driving cars violate traffic rules to avoid accidents (e.g., to cross into the opposing traffic lane to avoid hitting a parked car).
- Should self-driving cars continue on a yellow light to avoid suddenly stopping and getting rear-ended.
- If harm is unavoidable, should self-driving cars protect the young over the old.
- There are ethical concerns about the data being collected by such cars and potential for them being hacked.

**Please respond to the attention check questions below based on the the above information**

**--> You can only move ahead after answering ALL the questions below accurately and they are validated.**

**Please respond to the following questions about self-driving cars**

**If you were to consider adopting or buying a self-driving car, how important are each of the following benefits  to you.**

**If you were to consider adopting or buying a self-driving car, how important is it that the following technical challenges are overcome.**

**If you were to consider adopting or buying a self-driving car, how important is it that the following human or legal challenges are overcome.**

**If you were to consider adopting or buying a self-driving car, how important is it that the following ethical challenges are overcome.**

**Earlier you read about different challenges - technical, legal, and ethical - for self-driving cars.**

**Now please consider all these challenges together and rate their relative importance.**

**--> Please allocate 100 points to indicate relatively how important are each of these challenges below for self-driving cars.**

**--> The more important a challenge to overcome - in your opinion - the more points you allocate to that challenge.**

**--> Note that some challenges may be more rarely occurring events than others but you are the judge of whats important to you.**

## **[The TOTAL points you allocate across all these challenges below should add up to 100.]**

**Please respond to some more questions about self-driving cars**

**Background**

Now please provide some general information below.

Finally, we'd like to know a little bit about your background. This will help us compare responses from many different participants and ensure that we have a representative sample for our study.

**Follow-up Questions (Study 1)**

Imagine that it is the future, and self-driving cars are now available in the market.

--> These cars can communicate with and respond to users using a voice-based interface.

These self-driving cars have overcome all the technical challenges identified earlier and a robust legal policy and framework has been developed to handle any issues of liability and damages.

--> Only one issue remains, which is the Ethical Issue: how should self-driving cars decide between protecting pedestrians versus passengers in cases where harm is unavoidable and one of these parties will be harmed.

--> An overall ethical rule (setting) is required to program self-driving cars.

As such, self-driving cars can be programmed with one of the following ethical settings:

Ethical Setting A: Priority to protect the life of passengers over that of pedestrians from harm.

Ethical Setting B: Priority to protect the life of pedestrians over that of passengers from harm.

Ethical Setting C: Randomly choose between protecting the life of pedestrians versus that of passengers from harm.

***OTHERS* condition**

Imagine that self-driving cars are available in the market, and the manufacturers are seeking your

opinion about the above ethical settings for self-driving cars.

Please provide your opinion below about the ethical settings for self-driving cars.

Which of the following ethical setting should be programmed into self-driving cars?

***Self-BUY* condition**

Imagine that you decide to buy and OWN a self-driving car as shown above for regular use.

Please provide your opinion below about the ethical setting you would want for the self-driving car you buy and OWN for your regular use.

***Self-Buy Visible* condition**

Imagine that you decide to buy and OWN a self-driving car as shown above for regular use.

--> As a part of the government regulations it has been mandated that for ALL self-driving cars their chosen ethical setting will be VISIBLE to everyone.

Specifically, the above settings will be displayed on top of each car with a lighted box in following colors

Ethical Setting A: in color RED

Ethical Setting B: in color GREEN

Ethical Setting C: in color YELLOW

Now, please provide your opinion below about the ethical setting you would want for the self-driving car you buy and OWN for your regular use.

***Self-Rent* condition**

Imagine that you decide to RENT a self-driving car as shown above, which is available on a pay-per-use basis.

Please provide your opinion below about the ethical setting you would want for the self-driving car you RENT and pay-per-use as you need.

***Self-Buy Norm* condition**

Imagine that you decide to buy and OWN a self-driving car as shown above for regular use.

Please provide your opinion below about the ethical setting you would want for the self-driving car you buy and OWN for your regular use.

***Self-Buy Default* condition**

Imagine that you decide to buy and OWN a self-driving car as shown above for regular use.

Please provide your opinion below about the ethical setting you would want for the self-driving car you buy and OWN for your regular use.

**Supplementary Tables**

**Table S1: Regression Analysis: Purchase Intention for AVs Based on Benefits, Relative Importance of Different Challenges, and Control Measures (Study 1).**

| *Measure* | | *Coefficient* | | | *Std. Error* | | | *t* | | *p* | | |  |
| --- | --- | --- | --- | --- | --- | --- | --- | --- | --- | --- | --- | --- | --- |
| (Intercept) |  | | 0.435 |  | | 0.502 |  | | 0.866 | |  | 0.386 |  |
| Benefits_freeup_commute_time |  | | 0.335 |  | | 0.045 |  | | 7.508 | |  | < .001 |  |
| Prior_Exposure_AV_research |  | | 0.220 |  | | 0.039 |  | | 5.639 | |  | < .001 |  |
| Benefits_mobility_elderly_disbaled |  | | 0.153 |  | | 0.046 |  | | 3.308 | |  | < .001 |  |
| Benefits_reduce_health_costs |  | | 0.142 |  | | 0.052 |  | | 2.727 | |  | 0.006 |  |
| Driving_Experience |  | | -0.198 |  | | 0.068 |  | | -2.904 | |  | 0.004 |  |
| Education |  | | 0.155 |  | | 0.046 |  | | 3.381 | |  | < .001 |  |
| Benefits_reduce_traffic_congestion |  | | 0.119 |  | | 0.057 |  | | 2.085 | |  | 0.037 |  |
| Age |  | | -0.007 |  | | 0.003 |  | | -2.273 | |  | 0.023 |  |
| Importance_Ethical_dilemma_pass_ped |  | | -0.019 |  | | 0.009 |  | | -2.052 | |  | 0.040 |  |
|  |  | |  |  | |  |  | |  | |  |  |  |
|  |  | |  |  | |  |  | |  | |  |  |  |

*Note.*  The above is the final model in the stepwise regression (*adj.R^2^* = .221). The following covariates were considered but not included: Income, Political_Orientation, Importance_Tech_innercity, Importance_Tech_weather, Importance_Tech_recognize_objects, Importance_Tech_recognize_gestures, Importance_Legal_liability_harm, Importance_Legal_who_pays_fines, Importance_Ethical_data_privacy, Importance_Ethical_protect_young_old, Benefits_reduce_car_accidents, Benefits_reduce_insurance_costs.

**Table S2: Relative Risk of the Ethical, Legal and Technical Issues for AVs (Study 2)**

| *Measure* | *N* | | *Mean* | | *SD* | |  |  |
| --- | --- | --- | --- | --- | --- | --- | --- | --- |
| Risk_Ethical_dilemma_pass_ped |  | 672 |  | 15.607 |  | 7.252 |  |  |
| Risk_Tech_innercity |  | 672 |  | 9.289 |  | 8.897 |  |  |
| Risk_Tech_weather |  | 672 |  | 9.716 |  | 5.279 |  |  |
| Risk_Tech_recognize_objects |  | 672 |  | 10.561 |  | 5.662 |  |  |
| Risk_Tech_recognize_gestures |  | 672 |  | 10.124 |  | 4.801 |  |  |
| Risk_Legal_liability_harm |  | 672 |  | 9.696 |  | 5.127 |  |  |
| Risk_Legal_who_pays_fines |  | 672 |  | 8.524 |  | 5.046 |  |  |
| Risk_Ethical_yellow_light |  | 672 |  | 7.546 |  | 4.082 |  |  |
| Risk_Ethical_data_privacy |  | 672 |  | 8.005 |  | 4.626 |  |  |
| Risk_Ethical_protect_young_old |  | 672 |  | 10.932 |  | 5.788 |  |  |

**Table S3: Relative Risk of AV Ethical Dilemma versus Other Issues (Study 2)**

|  | | | | | | | | | | | | |
| --- | --- | --- | --- | --- | --- | --- | --- | --- | --- | --- | --- | --- |
| *Measure 1* | |  | | *Measure 2* | | *t* | | *df* | *p* | |  |  |
| Risk_Ethical_dilemma_pass_ped |  | - |  | Risk_Tech_innercity |  | 12.482 |  | 671 |  | < .001 |  |  |
| Risk_Ethical_dilemma_pass_ped |  | - |  | Risk_Tech_weather |  | 15.484 |  | 671 |  | < .001 |  |  |
| Risk_Ethical_dilemma_pass_ped |  | - |  | Risk_Tech_recognize_objects |  | 13.426 |  | 671 |  | < .001 |  |  |
| Risk_Ethical_dilemma_pass_ped |  | - |  | Risk_Tech_recognize_gestures |  | 15.023 |  | 671 |  | < .001 |  |  |
| Risk_Ethical_dilemma_pass_ped |  | - |  | Risk_Legal_liability_harm |  | 16.349 |  | 671 |  | < .001 |  |  |
| Risk_Ethical_dilemma_pass_ped |  | - |  | Risk_Legal_who_pays_fines |  | 18.916 |  | 671 |  | < .001 |  |  |
| Risk_Ethical_dilemma_pass_ped |  | - |  | Risk_Ethical_yellow_light |  | 24.414 |  | 671 |  | < .001 |  |  |
| Risk_Ethical_dilemma_pass_ped |  | - |  | Risk_Ethical_data_privacy |  | 20.966 |  | 671 |  | < .001 |  |  |
| Risk_Ethical_dilemma_pass_ped |  | - |  | Risk_Ethical_protect_young_old |  | 14.889 |  | 671 |  | < .001 |  |  |
|  | | | | | | | | | | | | |
| *Note.*  Student's t-test based on pair-wise mean comparisons. | | | | | | | | | | | | |

**Supplementary Figures**

**Fig. S1. (a) Relative importance of the challenges in the design and operation of AVs based on prior exposure to AV dilemmas (study 1).** Participants (*n* = 1,006) were asked to allocate 100 points among the 10 listed ethical, legal and technical issues based on the perceived importance to overcome these issues. Split based on prior exposure (no / yes) to AV dilemmas.

**(b) Relative risk in the issues in the design and operation of AVs based on prior exposure to AV dilemmas (study 2).** Participants (*n* = 672) were asked to allocate 100 points among the 10 listed ethical, legal and technical issues based on how much risk they perceived in these issues. Split based on prior exposure (no / yes) to AV dilemmas.

**Fig. S2: Importance of the benefits and the perceived risks in the design and operation of AVs (study 2).** Participants (*n* = 672) rated how important were the listed benefits and how much risk they perceived in the listed technical, legal / human, and ethical challenges if they were to consider adopting an AV. All ratings were on 1(not at all)-to-7(very high) scales.

**Fig. S3. Ethical preferences for AVs (study 1 follow-up) by purchase interest.** Participants were asked how should AVs be programmed in a dilemma where harm was unavoidable: “protect passengers over pedestrians”, “protect pedestrians over passengers”, or “no priority”. They were randomly assigned to one of six conditions: (1) AVs in general (*others*), (2) AVs bought for own use (*self-buy*), (3) AVs for own use with default setting of no priority (*self-buy-default*), (4) AVs for own use stating that majority prefer protecting passengers or no priority (*self-buy-norm*), (5) AVs for own use with visible ethical settings (*self-buy-visib*), and (6) AVs rented for own use (*self-rent*).
